# Meristematic cell proliferation and ribosome biogenesis are decoupled in diamagnetically levitated Arabidopsis seedlings

**DOI:** 10.1186/1471-2229-13-124

**Published:** 2013-09-05

**Authors:** Ana Isabel Manzano, Oliver J Larkin, Camelia E Dijkstra, Paul Anthony, Michael R Davey, Laurence Eaves, Richard JA Hill, Raul Herranz, F Javier Medina

**Affiliations:** 1 Centro de Investigaciones Biológicas (CSIC), Ramiro de Maeztu 9, E-28040 Madrid, Spain; 2 School of Biosciences, University of Nottingham, Sutton Bonington Campus, Loughborough LE12 5RD, UK; 3 School of Physics & Astronomy, University of Nottingham, Nottingham NG7 2RD, UK; 4 Present Address: Faculty of Health and Life Sciences, Coventry University, Coventry CV1 5FB, UK

## Abstract

**Background:**

Cell growth and cell proliferation are intimately linked in the presence of Earth’s gravity, but are decoupled under the microgravity conditions present in orbiting spacecraft. New technologies to simulate microgravity conditions for long-duration experiments, with stable environmental conditions, in Earth-based laboratories are required to further our understanding of the effect of extraterrestrial conditions on the growth, development and health of living matter.

**Results:**

We studied the response of transgenic seedlings of *Arabidopsis thaliana*, containing either the CycB1-GUS proliferation marker or the DR5-GUS auxin-mediated growth marker, to diamagnetic levitation in the bore of a superconducting solenoid magnet. As a control, a second set of seedlings were exposed to a strong magnetic field, but not to levitation forces. A third set was exposed to a strong field and simulated hypergravity (2 *g*). Cell proliferation and cell growth cytological parameters were measured for each set of seedlings. Nucleolin immunodetection was used as a marker of cell growth. Collectively, the data indicate that these two fundamental cellular processes are decoupled in root meristems, as in microgravity: cell proliferation was enhanced whereas cell growth markers were depleted. These results also demonstrated delocalisation of auxin signalling in the root tip despite the fact that levitation of the seedling as a whole does not prevent the sedimentation of statoliths in the root cells.

**Conclusions:**

In our model system, we found that diamagnetic levitation led to changes that are very similar to those caused by real- [e.g. on board the International Space Station (ISS)] or mechanically-simulated microgravity [e.g. using a Random Positioning Machine (RPM)]. These changes decoupled meristematic cell proliferation from ribosome biogenesis, and altered auxin polar transport.

## Background

Plants have evolved over millions of years on Earth under the influence of gravity. As a consequence, this parameter plays an important role in plant development [1]. The main gravitropic response, namely “gravitropism”, not only modulates root and stem orientation during growth, but also modulates indirectly the developmental mechanisms of plants. A transduction cascade triggered by gravitropism, in which high density statoliths present in the columella cells play a major role, appears to affect the distribution of the plant hormone auxin [2,3] which is a key regulator in plant growth and development in response to environmental signals [4].

Alternative freefall techniques and facilities, such as drop towers, sounding rockets or parabolic flights, can be used to study the effects of altered gravity (both hypogravity and hypergravity). In these cases altered gravity is attained for a short duration, with alternating periods of hypergravity and microgravity. In an orbiting spacecraft such as the International Space Station, weightlessness is maintained for long periods of time, but access to the platform is severely constrained in terms of the number of experiments that can be performed, the availability of crew time for experimenta-tion and cost. These limitations have encouraged the development of ground-based systems to simulate modified gravity conditions, such as 2D-clinostats, Random Positioning Machines (RPMs), and adapted centrifuges. Here, we use an alternative ground-based technique, which exploits the force exerted on diamagnetic material when exposed to a strong and inhomogeneous magnetic field [5-7].

Alterations in environmental parameters produce changes in the processes of plant growth, differentiation and development. Environmental changes affect the whole plant through alterations to cellular processes and mechanisms, including cell proliferation and growth, which are basic and essential functions of cellular life. It is well known that signals transduced between different plant organs are capable of activating key modulators of cell growth and division in meristems; the reception of these signals and the response to them is termed “meristematic competence” [8]. Cell proliferation in the root meristem constitutes the source of cells for root growth and differentiation, together with the cellular basis for the developmental programme of the plant [9,10].

Previous experiments have demonstrated that the environmental conditions during spaceflight and in a clinostat cause changes in the duration of the cell cycle [11,12]. A series of experiments were carried out in the Spacelab IML-1 and IML-2 missions with lentil seedlings grown for 28-29 h, i.e. the time necessary to complete the first cell cycle after germination. The percentages of the different cell cycle phases (G1, S, G2 and M) were determined using Feulgen staining. The authors concluded that the cell cycle was slowed in microgravity [12,13]. However, it is important to take into consideration that the alterations may involve different effects, depending on the time of exposure to microgravity and/or on the phase of development of the plants or seedlings [14,15]. Other experiments have shown that cell proliferation parameters are modified in plants grown in space, including those of maize [16] and *Arabidopsis*[17], where there occurred a decoupling between cell proliferation and cell growth in the meristems of *Arabidopsis* seedlings grown for 4 days under these conditions.

Here we report the results of growing seedlings under diamagnetic levitation for 2 and 4 days from germination, together with controls grown in normal gravity (1 *g*). All of the analyses were performed on the three cellular layers of the cortical cylinder of the root meristem, namely the epidermis, cortex and endodermis [10]. These cells are specialised in proliferation, such that their only function is to grow and divide continuously at a rate three times greater than cells in the central cylinder [18]. In this zone of the root tip we analysed a series of cellular parameters which are reliable indicators of the status of cell growth and proliferation, in order to assess the variations in these functions. Regarding proliferation, we measured the number of cells per millimetre in meristematic cell layers (approx. 100 ± 30 μm from the root tip); we term the rate of increase of this parameter the “rate of local cell production” [19]. As an additional parameter of proliferation, we determined the expression levels of the cell cycle regulator gene cyclin B1, using a CYCB1:GUS transgenic plant line. We chose this transgenic construct because cyclin B1 is a key factor in the G2/M transition [20,21], and is potentially involved in the microgravity-related proliferation/growth decoupling [22]. In addition, we studied the pattern of auxin distribution using DR5:GUS transgenic seedlings; the staining pattern obtained with this reporter gene is an accurate indicator of auxin polar transport [23].

Cell proliferation depends on a continuous supply of proteins; this is the main requirement to reach the critical size necessary for cell division. Ribosomes, localised in the rough endoplasmic reticulum, are the protein factories of cells, and the rate of ribosome biogenesis is directly correlated, in proliferating meristematic cells, with cell growth necessary for cell division [24,25]. This means that, in meristems, cell growth is determined largely by the activity of RNA polymerase I, which controls ribosomal RNA synthesis and ribosome biogenesis in the nucleolus [26]. An unequivocal functional linkage exists between the rate of ribosome biogenesis and the nucleolus ultrastructure. This linkage has been demonstrated in several cellular model systems [27,28], in particular in plants [29-31], using cellular models characterised by high cell proliferation activity. Therefore, in order to obtain an accurate estimate of the rate of ribosome biogenesis, we determined the nucleolar size and the relative amount and distribution of nucleolar subcomponents, such as fibrillar centres (FCs) and the granular component (GC). In addition to these morpho-functional data on the nucleolus, the efficiency of the process of ribosome biogenesis was assessed by determining variations in the levels of nucleolin, the major nucleolar protein of actively proliferating cells, identified in animals, plants and yeast as playing a key role in different steps of the synthesis and processing of pre-ribosomal precursors [28,31]. All these data have been correlated with those obtained from cell proliferation parameters.

## Methods

### Diamagnetic levitation

**Figure 1:**
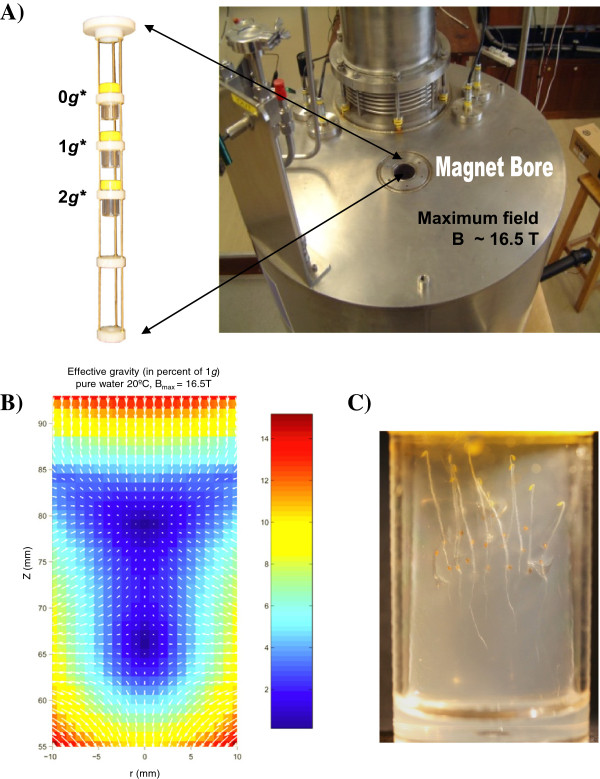
**Experimental set up and seedling growth. A)** Three samples were exposed simultaneously to the magnetic field, in three different positions within the magnet bore: 0 g*, 1 g* and 2 g* tubes. An additional sample was maintained under the same conditions, except for the magnetic field, outside the magnet (1 *g* control). **B)** Effective gravity acting on water within the 0 g* tube. The colour scale indicates the magnitude of the effective gravity as percent of the ground gravity *g*. Arrows show the magnitude and direction of the effective gravity vector. **C)** Image taken of the sample tube located at the 0 g* position, after the 4-day experiment. After growing in darkness, seedlings show a clear orientation according to the gravity vector.

For levitation of a biological organism, a strong, spatially-varying magnetic field, such as that produced by a dissipative Bitter-type or superconducting solenoid magnet, is required [32-35]. Our experiments were performed using an Oxford Instruments superconducting solenoid magnet with a room-temperature vertical bore, located at the University of Nottingham, UK. We used the magnet to simulate aspects of altered gravity conditions: microgravity and hypergravity (Figure 1A).

Inside the bore, the magnetic field is strongest at the centre of the solenoid. Inside the upper section of the bore, diamagnetic material is pushed upward by the induced magnetic force, away from the strong magnetic field near the centre, so that it opposes and balances the gravitational force. In the lower section, diamagnetic material is repelled downwards in the same direction as gravity. The vertical repulsive force on an object is proportional to the product of four terms: the magnitude of the magnetic field *B;* its vertical gradient ∂*B*/∂*z*; the mass of the object and its magnetic mass susceptibility χ_m_[35,36]. The net force (gravitational plus magnetic force) per unit mass is the effective gravity, g*, acting on the material. That the net force can be expressed conveniently in terms of an effective gravity follows from the fact that the diamagnetic force is a body force, i.e. it acts throughout the body of the object, as does the gravitational force. In contrast, the buoyancy force on a body in a fluid cannot be expressed this way, since it acts only on the submerged surface of the body.

In our experiments, we used a magnetic field at the geometric centre of the solenoid of 16.5 T, which allows a water droplet to levitate in stable mechanical equilibrium approximately 80 mm above the centre of the solenoid (Figure 1B). The technique of stable diamagnetic levitation has been described in detail elsewhere, e.g. [5,34,36]. Seedlings and imbibed seeds of *Arabidopsis* levitated in the same position in the magnet as the water droplet, since the magnetic mass susceptibility of most of the plant tissues is similar to that of water [37]. Under these conditions, the gravitationally-induced stresses on such tissues are expected to be much reduced by diamagnetic levitation [35]. One cellular component that is *not* levitated under these conditions is the starch-rich statolith, which, in contrast with most other tissues, has a |χ_m_| that is significantly smaller than that of water. Although the force of gravity on the statolith is reduced substantially by the high gradient magnetic field, the statoliths still sediment under the residual gravitational force, albeit at a reduced rate. The movement of these specialised amyloplasts within the cell, under the action of gravity, is one of the proposed cellular mechanisms for sensing the direction of gravity [38].

We use the label 0 g* to refer to the point in the magnetic field where water levitates stably. The asterisk indicates that ‘0 *g*’ refers to the effective gravity acting on *pure water* at this point, and also serves as a reminder that a strong magnetic field is present. Note that this label does not necessarily imply that the effective gravity acting on the *plant tissue* at this point is exactly zero. We label the geometric centre of the solenoid as the 1 g* point; here there is no gradient in the magnetic field, and therefore no diamagnetic force, so the effective gravity acting on water is 1 *g*. We label a third point, 80 mm below the centre of the field, as the 2 g* point. Here, the effective gravity on water is 2 *g*. The magnetic field at the 0 g* and 2 g* points is 11.5 T, and 16.5 T at the 1 g* point.

### Cultivation of seeds and their positioning within the magnet bore

Seeds of *Arabidopsis thaliana* (L.), Heynh., ecotype “Columbia” (Col-0) were used in these experiments. The seeds carried either the CYCB1:GUS reporter gene construct [39] or the DR5:GUS reporter gene construct [23], enabling *in situ* measurements of the expression of the cyclin B1 gene, or of the distribution of auxin, respectively. These constructs were kindly supplied by Dr. E. Carnero-Diaz (UPMC, Paris, France). The seeds were sterilised in 1.25% (v/v) sodium hypochlorite and 1% (v/v) Triton X-100 for 10 min and then rinsed in sterile water. For each sample, seeds were then placed on the surface of an agar slant [containing 0.5% (w/v) agar with MS plant culture medium ([40]; Duchefa) in a 25 mm-diameter, 55 mm-tall plastic tube. Twenty seeds were loaded into each tube which was then maintained at 4°C for two days in a refrigerator.

Four experimental conditions were investigated, within four tubes. After removal from the refrigerator, the first tube was positioned in the magnetic field such that the centre of the tube was located at the 0 g* point in the field. Henceforth, we refer to this tube as the 0 g* tube. The effective gravitational force acting on water did not exceed 3 × 10^-2^ *g* for any of the seedlings (Figure 1B). A second group of seedlings, similarly prepared, were positioned in the magnetic field to enclose the 1 g* point. A third tube of seedlings was placed to enclose the 2 g* point in the field, while a control experiment (1 *g*) was run in a fourth tube outside the magnet, in a temperature-controlled incubator. The seeds were maintained at 24°C in the magnet, and in the incubator, and allowed to germinate in the dark. The arrangement of seeds in the 1 g* and 2 g* tubes replicated the arrangement in the 0 g* tube. The experiments in the 0 g*, 1 g*, 2 g* and 1 *g* tubes were run simultaneously. After either two or four days’ growth in the dark, specimens were removed promptly from the tubes, photographed and plunged into a fixative solution (see below). The time that elapsed between removal of the first sample from the magnet and fixation of the last one did not exceed 20 min.

### Sample processing for CycB1:GUS and DR5:GUS analyses

For GUS analysis, samples were fixed in 90% acetone at −20°C for 24 h. Specimens were washed with 100 mM phosphate buffer. The GUS signal was revealed by enzymatic reaction (5 mM potassium ferrocyanide and ferricyanide, 100 mM phosphate buffer and 40 mM X-Glc) in the dark. Seedlings were washed and mounted on 8 mm 8-well slides and observed with a Leica DM2500 microscope. Images were recorded digitally using a Leica DFC320 CCD camera and were processed using the QWin Standard (Leica Microsystems) and Image J 2.0 (imagejdev.org) software packages. The integrated optical density (IOD) was calculated from the stained area multiplied by the Optical Density (OD) in blue light. An unstained zone of the root tip was used for the control (background optical density).

### Sample processing for morphometric and ultrastructural analyses

Samples were fixed in 4% (w/v) paraformaldehyde for 2 hours at room temperature for the morphometric and immunocytochemical studies, and in 3% (w/v) glutaraldehyde for 1 hour at room temperature for ultrastructural microscopy studies. They were then washed 3 times in phosphate buffered saline (PBS), 10 min each, dehydrated in an ethanol series, treated for the methylation-acetylation procedure [41] and finally embedded in LR White resin (London Resin Co., UK).

### Microscopy, immunocytochemistry and quantification

From resin-embedded materials, 2 μm thick (semithin) sections were obtained and observed unstained, under a Leica DM2500 phase contrast microscope. Images were recorded digitally with a Leica DFC320 CCD camera. For ultrastructural studies, 60–90 nm ultrathin sections were mounted on Formvar-coated nickel grids, stained with uranyl acetate and lead citrate and observed using a Jeol 1230 electron microscope, operating at 100 kV.

Measurements of seedling and root length, number of cells per mm in root cell rows, cross-sectional area of nucleoli, and proportion of nucleolar granular component (GC) were obtained from the digital images using the quantitation software “QWin Standard” (Leica Microsystems, Germany).

Measurements of cell parameters were made on root meristematic cells belonging to the epidermal, cortical and endodermal layers of the root. Twenty seedlings were used in measurements of seedling length; 10 roots were analysed for the number of cells per mm of root length; the cross-sectional areas of 60 nucleoli were measured using optical microscopy; 15 nucleoli were analysed for GC proportion using electron microscopy, and a further 15–20 nucleoli were used for immunocytochemistry analysis. For immunogold labelling, ultrathin sections were incubated with an anti-nucleolin polyclonal antibody [42] (kindly supplied by Dr. J. Sáez-Vásquez, Perpignan, France), diluted 1:100 for 90 min at room temperature, followed by goat-anti rabbit IgG coupled with 12 nm colloidal gold particles (Jackson Immuno-research Laboratory, USA) diluted 1:50 for one hour at room temperature. Statistical analysis of the data was performed using Statgraphic 5.1 software (Statpoint Technologies, INC, USA). Quantitative variables were assessed using mean and standard deviation values after checking normality with the Kolmogorov–Smirnov test. Mean values were compared using the Student t-test for independent samples; differences were considered significant for a bilateral value lower than 0.05.

## Results

### Growth of seedlings in the different positions in the magnet bore

**Figure 2:**
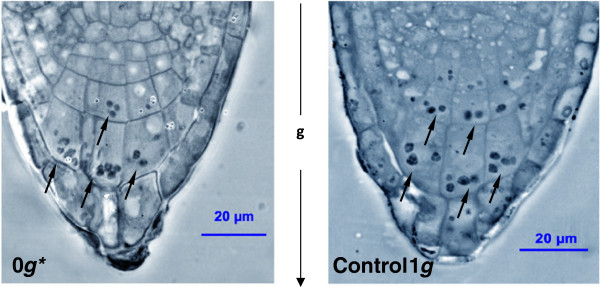
**The root columella cells observed by phase-contrast microscopy.** (Left-hand image) Samples were fixed, embedded in resin and 2 μm semithin sections were obtained from them and observed under phase-contrast light microscopy. Those samples grown under magnetic levitation (0 g* tube) for 4 days show the same distribution of statoliths (indicated by arrows) as in control 1 *g* samples (right-hand image) grown outside the magnet. The statoliths collect near the basal membrane of the columella cells, under the force of gravity; the arrow between the images indicates the direction of gravity.

For all seedlings grown within the magnet, whatever their position in the magnetic field, roots grew downward, i.e. towards the ground (Figure 1C). Thus, diamagnetic levitation of the cellular components with magnetic mass susceptibility close to that of water was not sufficient to suppress the gravitropic response. Columella cells, which are specialised in detecting the gravity vector and establishing the root growth direction, were observed by phase-contrast microscopy (Figure 2). The statoliths of columella cells were found in the same position both in samples from the 0 g* tube and in samples from the 1 *g* control external to the magnet. This indicated that the magnetic field used in these experiments was not capable of suspending the starch grains within the cell.

### Morphometric analysis of the seedlings and root meristems

Seedling length after 2 and 4 days of growth was used as the first parameter for estimating cell growth and proliferation. After 2 days’ growth no significant differences were found in seedling length between the internal and external controls (1 *g* and 1 g* tubes). However, significant differences were found between samples grown in the 0 g* tube and those grown in the 2 g* tube. Seedlings grown in the 0 g* tube were shorter than those grown under normal gravity conditions, whereas the conditions in the 2 g* tube produced an increase in the length of the seedlings. Differences were also observed between samples grown in these tubes and those grown in both control conditions (1 *g* and 1 g*).

**Figure 3:**
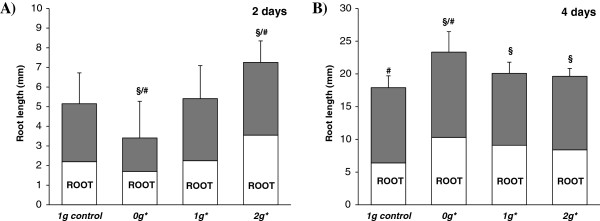
**Morphometric parameters of seedlings.** Diagram of seedling and root length in the two-day **(A)** and four-day long **(B)** experiments. Statistically significant differences in length (p < 0.05), compared to the control, are indicated with a ‘§’ symbol when compared with the 1 *g* external control, and with ‘#’ when compared with the 1 g* control. The most pronounced differences were found in the 0 g* sample, which shows significant differences with respect to both external and internal controls in the two experiments. Seedlings in the internal and external controls (1 *g* and 1 g*) show significant differences after 4 days’ growth, but not after 2 days. After 4 days, all samples grown within the magnet (0 g*, 1 g* and 2 g*) are significantly different from the external control (1 *g*).

Seedlings grown for 4 days in the 0 g* tube were significantly longer than in both 1 *g* and 1 g* controls. However, samples from all three tubes within the magnet, including the 2 g* and 1 g* tubes, showed an increase in seedling length compared to the external 1 *g* control (Figure 3).

Additional parameters related to cell growth and proliferation were evaluated in 2 μm semithin sections of root meristems obtained from all samples, and observed by phase-contrast microscopy. The cortical meristem cells (epidermis, cortex and endodermis) were studied in a zone 50 ± 30 μm above the quiescent centre (approx. 100 ± 30 μm behind the root tip), where the meristem width (μm), local cell proliferation rate (number of cells per mm in each cell row) and nucleolar size (area in μm^2^) were quantified.

**Figure 4:**
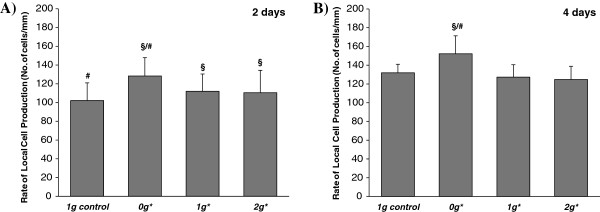
**Rate of local cell production (Number of cells/mm in each cell row of the root meristem). A)** Two-day experiment. **B)** Four-day experiment. Statistically significant differences in cell production (p < 0.05), compared to the control, are indicated with a ‘§’ symbol when compared with the 1 *g* external control, and with ‘#’ when compared with the 1 g* control. Seedlings grown in the 0 g* tube show consistently higher cell proliferation rates compared with controls.

In a preliminary report of similar experiments performed by centrifugation, it was observed that root width at the level of the meristem was greater in hypergravity conditions compared to 1 *g* controls [43]. Detailed observations using semithin sections of the centre of root tips corroborated this effect, although the differences between 2 g* and 1 *g* samples were smaller than expected (Additional file 1: Figure S1). The rate of local cell production was quantified by counting the number of cells per unit length in the cortical cylinder cell rows, to give an estimate of the proliferation rate. A significant increase in the proliferation rate was observed in samples in the 0 g* tube, for both 2-day and 4-day experiments. Moreover, in the 4-day experiment, cell proliferation rate increased significantly in all tubes in the magnet (0 g*, 1 g* and 2 g*) compared to the 1 *g* control (Figure 4).

**Figure 5:**
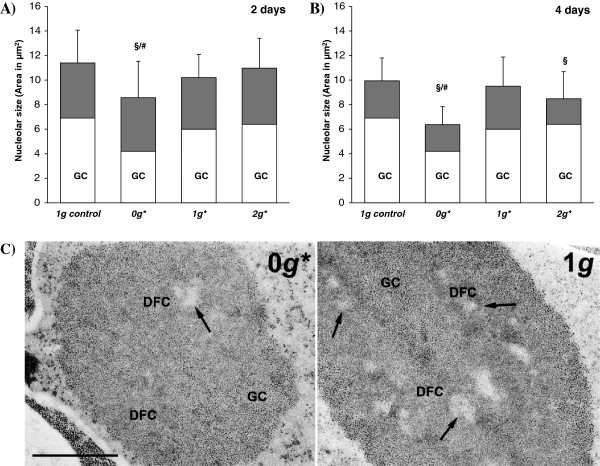
**Average size of the nucleolus (cross-sectional area in μm**^**2**^**) and percentage of granular component (GC). A)** Two-day experiment. **B)** Four-day experiment. Statistically significant differences in nucleolar size (p < 0.05), compared to the control, are indicated with a ‘§’ symbol when compared with the 1 *g* external control, and with ‘#’ when compared with the 1 g* control. Seedlings grown in the 0 g* tube show consistently a smaller nucleolus size. **C)** Representative electron microscope images of nucleoli from the 4-day-long experiment. Even though the nucleolus is clearly smaller in samples from the 0 g* tube, compared to the external control, the proportion and distribution of the different ultrastructural components of the nucleolus is similar in both conditions. GC: granular component. DFC: dense fibrillar component. Arrows indicate fibrillar centres.

Nucleolar size was quantified in order to obtain an estimate of nucleolar activity (i.e. the rate of ribosome production) and, consequently, of cell growth. No significant differences in nucleolar size were found between samples from the 1 g* tube and samples from the 1 *g* external control. At both 2 and 4 days after germination, a reduction was observed in the nucleolus size in samples from the 0 g* tube compared to both control conditions (1 g*/1 *g*). A reduction in the nucleolus size of samples grown in the 2 g* tube was found after 4 days’ of growth, but not after two days (Figure 5).

### Ultrastructural and immunocytochemical studies in meristematic cells

An additional estimate of the changes in cell growth was obtained from the ultrastructure of the nucleolus in the cells of the cortical cylinder. Some ultrastructural parameters of the nucleolus of highly proliferating root meristematic cells are functionally associated with the rate of ribosome biogenesis in these cells [44]. These parameters include the nucleolar size (which was quantified in semithin sections, using light microscopy), the relative proportion and distribution of the nucleolar subcomponents, and the levels of the nucleolar protein, nucleolin. Fixation in glutaraldehyde was necessary for clear dis-crimination of nucleolar subcomponents. These analyses did not reveal any difference in the proportion or distribution of the internal components of the nucleolus (Figure 5C).

**Figure 6:**
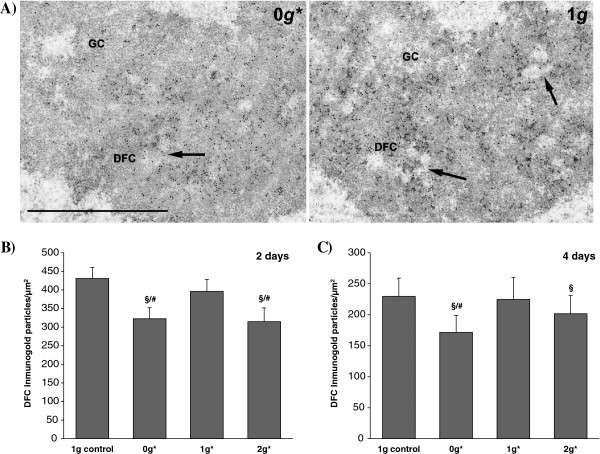
**Immuno-gold electron microscopical detection of the nucleolar protein nucleolin. A)** Portions of nucleoli from root meristematic cells, labelled with anti-nucleolin antibody and visualised with colloidal gold particles, are shown for the 2-day experiment: (left) 0 g* samples, (right) external 1 *g* control samples. Nucleolin is localised in the dense fibrillar component (DFC), surrounding fibrillar centres (arrows) in both cases. The 0 g* sample shows a lower density of gold particles, indicating a lower nucleolus activity. **B** and **C)** Density of immunogold particles measured after two- **(B)** and four-day long **(C)** experiments. Statistically significant differences in density (p < 0.05), compared to the control, are indicated with a ‘§’ symbol when compared with the 1 *g* external control, and with ‘#’ when compared with the 1 g* control.

Immunogold localisation of nucleolin was carried out on paraformaldehyde-fixed samples. In all cases, nucleolin was located in the dense fibrillar component (DFC) of the nucleolus, surrounding fibrillar centres (FCs), where it has been reported to be located in active nucleoli of plant meristematic cells [44] (Figure 6).

Nucleolin quantification using immunocytochemical labelling was performed in seedlings grown for 2 and 4 days within the magnet. Samples from both 0 g* and 2 g* tubes showed less of this protein in their nucleoli, compared with samples from the 1 *g* and 1 g* tubes. Neither internal nor external controls (1 g* and 1 *g*) showed significant differences (Figure 6).

### Regulation of cell cycle progression, determined by the expression levels of Cyclin B1, estimated by the GUS reporter gene

**Figure 7:**
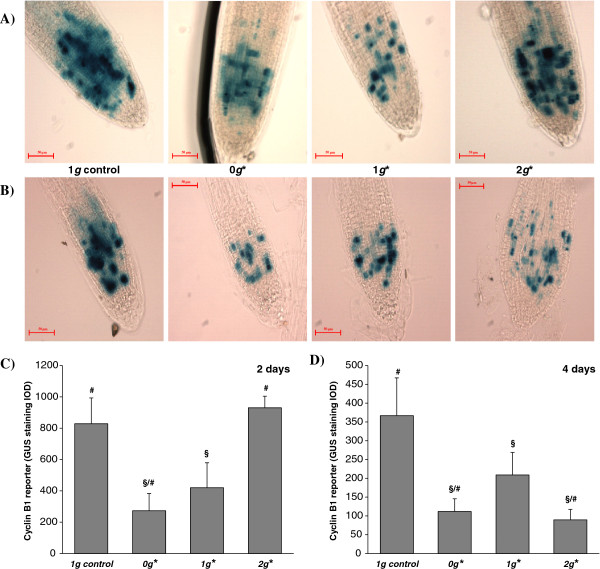
**Cyclin B1 expression in root tips revealed by GUS staining.** The use of the reporter gene line CYCB1:GUS allowed the microscopical visualization of the expression of the cyclin B1 gene. Whole mount preparations of roots were stained and observed by optical microscopy. **A)** 2 days’ and **B)** 4 days’ growth; from left to right: 1 *g* external control, 0 g*, 1 g* and 2 g*. **C)** and **D)** Quantitative study of the expression of the cyclin B1 gene by measuring the integrated optical density (I.O.D.) in the two-day **(C)** and in the four-day **(D)** experiments. Statistically significant differences in IOD (p < 0.05), compared to the control, are indicated with a ‘§’ symbol when compared with the 1 *g* external control, and with ‘#’ when compared with the 1 g* control. GUS staining show an overall decrease in the expression of this gene (which is usually considered to be a marker of cell proliferation)in the magnet. This is especially clear in samples from the simulated microgravity position (0 g*).

The activity of the cyclin B1 gene in seedlings carrying the CYCB1:GUS construct [39] was quantified by taking integrated optical density measurements of GUS-stained samples (Figure 7). As previously indicated, cyclin B1 is a key regulator of the G2/M transition of the cell cycle, and this gene is expressed specifically in G2, indirectly indicating the rate of entry into mitosis; since the product is specifically destroyed in anaphase, expression of the cyclin B1 gene is considered a good marker of cell division [45].

Seedlings grown in the 0 g* and 1 g* tubes showed a significant decrease in the expression of cyclin B1 (CYCB1;1) compared to the control samples grown outside the magnet (1 *g*), after both 2 and 4 days’ growth (Figure 7 and Table 1). The decrease of the expression of this gene in root meristematic cells was more pronounced in 0 g* samples than in 1 g* samples. The influence of the 2 g* conditions on the expression of this gene is less clear: after 2 days’ growth in 2 g*, measuremenents of cyclin B1 expression were similar to measurements taken from the control samples outside the magnet (1 *g*), but after 4 days’ growth, the gene expression is similar to that in 0 g* samples.

**Table 1:** **Summary of the changes observed in CYCB1:GUS seedlings exposed to levitation (0** g*) and hypergravity (2 g*) with respect to controls external (1 ***g*) and internal to the magnet (1** g*)

**Parameter**	**No. days post germination**	**0** g* with respect to :		**1** g* w.r.t. :	**2** g* w.r.t. :	
		**1** ***g *ctrl.**	**1** g* ctrl.	**1** ***g *ctrl.**	**1** ***g *ctrl.**	**1** g* ctrl.
Seedling length	2 days	**-**	**-**	**=**	**+ +**	**+ +**
Cell proliferation rate		**+ +**	**+**	**+**	**+**	**=**
Ribosome biogenesis (Nucleolar size)		**- -**	**- -**	**=**	**=**	**=**
Ribosome biogenesis (Nucleolin level)		**- -**	**- -**	**=**	**- -**	**-**
Cyclin B1 expression		**- -**	**-**	**-**	**=**	**+ +**
Seedling length	4 days	**+ +**	**+**	**+**	**+**	**=**
Cell proliferation rate		**+ +**	**+ +**	**=**	**=**	**=**
Ribosome biogenesis (Nucleolar size)		**- -**	**- -**	**=**	**-**	**=**
Ribosome biogenesis (Nucleolin level)		**- -**	**- -**	**=**	**-**	**=**
Cyclin B1 expression		**- -**	**-**	**-**	**- -**	**-**

Statistically significant differences with respect to controls are indicated with ‘+’ (increase) or ‘–‘(decrease) when p < 0.05, and with ‘+ +’/’- -‘when p < 0.01. ‘=’ indicates an insignificant difference.

### Auxin distribution, determined by the expression levels driven by the DR5 synthetic promoter, estimated by GUS reporter staining, in the root meristem

Auxin distribution was studied using a synthetic reporter construct with a DR5 promoter driving β-glucuronidase activity. This promoter contains 7 tandem repeats of an auxin-responsive TGTCT element, which has been shown to respond rapidly and specifically to active auxins, as many early auxin response genes do, by binding auxin response factors (ARFs) [46-48].

**Figure 8:**
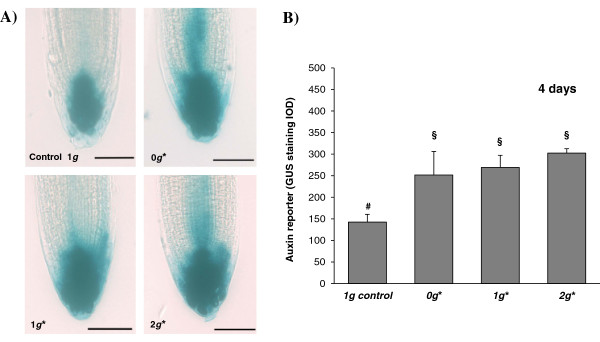
**Auxin distribution in root tips revealed by GUS staining.** The use of the reporter gene line DR5:GUS allowed the microscopical visualization of the auxin distribution. Whole mount preparation of roots were stained and observed by optical microscopy. **A)** Optical microscopy images of DR5-GUS-stained root meristems from seedlings grown for 4 days in the magnet; the staining shows the distribution of auxin in the root tip. From top-left to bottom right: samples from the *1 g* external control, 0 g*, 1 g* and 2 g* positions in the magnet. **B)** Quantitative study of the GUS staining by measuring the integrated optical density (I.O.D.) in the four-day experiment. Statistically significant differences in IOD (p < 0.05), compared to the control, are indicated with a ‘§’ symbol when compared with the 1 *g* external control, and with ‘#’ when compared with the 1 g* control.

The distribution of the staining pattern in this transgenic line had been demonstrated to reflect with high accuracy either the normal occurrence of the auxin polar transport, or its disruption, in the case that specific inhibiting drugs were used [46-48]. In our experiments under Earth’s gravity, outside the magnet, auxins were found to be located in the columella initial cells, with reduced concentration in the quiescent centre. However, in all samples exposed to the strong magnetic field (0 g*/1 g*/2 g*), both the intensity and the distribution of the DR5:GUS signal was altered, with the DR5:GUS activity being extended to the lateral layers, including at least part of the meristematic and stele cells (Figure 8). This distribution of the staining pattern corresponds to at least a partial inhibition of auxin polar transport, as demonstrated previously using specific inhibitors [46,48].

Table 1 summarises the parameters that we have used to evaluate cell proliferation and cell growth of seedlings, and our corresponding findings.

## Discussion

Altering the force of gravity modifies essential characteristics of root meristem activity such as the rate of cell proliferation and cell growth, which are cellular processes that appear strictly coordinated in meristematic cells (“meristematic competence”) [8]. An experiment carried out in real microgravity on a space mission has demonstrated that some parameters associated with cell proliferation or cell growth were altered in opposite directions, compared to corresponding values in the ground control experiment. The results of that work strongly suggested that the two cellular processes were uncoupled by microgravity: cell proliferation was enhanced while cell growth was inhibited, thus disrupting meristematic competence [17]. Here, we have built on those earlier experiments, and provided further support for their conclusions, in ground-based experiments simulating aspects of microgravity conditions by diamagnetic forces.

Diamagnetic levitation requires the application of strong magnetic fields. Some of our results suggest that the strong magnetic field may affect the seedlings even in the absence of the vertical field gradient required for levitation. Samples from the 1 g* tube (strong magnetic field, no vertical field gradient), where the effective gravity is 1 *g*, exhibited slight increases in cell proliferation rate at 2 days after seed-soaking (but not after 4 days) and slight increases in seedling length 4 days after cell soaking (but not after 2 days). The evidence is not strong enough to show conclusively a magnetic field effect on these parameters; further experiments would be required to confirm these effects. Some researchers claim that relatively low-intensity magnetic fields exert a ‘positive’ effect on seedlings, increasing rates of seed germination and growth [49-51]. Maize seeds germinated in a magnetic field of 125–250 mT (a magnetic field of order 100 times weaker than in our experiments), are claimed to produce an increase in germination rate and seedling length [52]. Some studies have claimed that weak magnetic fields of 0.5 mT (of order 10 times Earth’s magnetic field) enhances *Arabidopsis thaliana* seedling growth, but this was later shown not to be reproducible [53].

We obtained stronger evidence that the high magnetic field caused auxin to accumulate in the root tip, as detected with the DR5:GUS construct. Since this effect was observed in the 1 g* tube (at both 2 days and 4 days after seed-soaking), the effect is independent of the gradient of the field, suggesting that the effect is not associated with altered gravity. Rather, the behaviour suggests that the strong magnetic field used in these experiments inhibited polar auxin transport: this pattern of auxin distribution is the same as that observed after drug-induced inhibition of the transport with N-naftalamic acid (NPA) or in the *eir1* mutant (note that the *EIR1* gene encodes transmembrane proteins capable of mediating cellular auxin efflux) [47,54]. This inhibition produces changes in progression of the cell-cycle [55]. Cells that accumulate an excess of auxin are not capable of differentiating. Consequently, they retain meristem competence for an excessive length of time. Furthermore, high local auxin concentration in the root tip causes over-expression of genes involved in regulating the cell cycle [47,55].

It is well-known that the regulation of the progression of the cell cycle depends on the expression of a large group of genes. These genes play fundamental roles in different cellular and molecular processes, such as cytoskeleton synthesis and assembly, proteolysis, transcription, protein phosphorylation, biosynthesis of a wide variety of molecules, secondary metabolic processes, hormonal response, organelle function and even direct control of the cell cycle. A significant number of these genes were found to be affected in previous experiments on *Arabidopsis* callus cultures exposed to strong magnetic fields, of similar strength to the field used in these experiments, using microarray techniques [56].

The addition of a vertical magnetic field gradient induces a vertical diamagnetic force on the sample (levitating the samples in the 0 g* tube and doubling the effective weight of the samples in the 2 g* tube) which further alters many of the cell growth and proliferation parameters we have measured here. Notably, however, the roots of seedlings at all positions in the magnet were positively geotropic and grew in the direction of the gravity vector. Root gravitropism is governed by organelles called statoliths, which are contained in columella cells at the root tip. The major component of statoliths is starch, which has a density approximately 1.5 times that of the cytoplasm. The buoyancy of the cytoplasm does not, therefore, balance the gravitational force on the starch-rich organelle. The position of the statolith within the cell depends on the net (buoyant plus gravitational) force on the statolith; this is the key factor for the establishment of the root direction, according to the starch-statolith theory of gravitropism [38,57,58]. A detailed microscopic analysis of the columella cells of seedlings grown in the 0 g* tube in the magnet, showed that the position of the statoliths was at the bottom of the cells, indistinguishable from the position observed in samples from the 1 *g* external control, and also from the positions of the statoliths in the other two other positions of the magnet bore (1 g* and 2 g*). This finding is not unexpected, because the experiment was set up to levitate only those cellular components with magnetic mass susceptibility similar to that of water. The position of the statoliths within the cell *can* be altered using high-gradient magnetic fields [55,56] but the magnitude of *B* x ∂*B*/∂*z* required to levitate the statoliths is 3–4 times as large as that required to levitate water. In these experiments, the intracellular water, water-soluble cellular components and other components of the cell with a magnetic mass susceptibility similar to that of water were levitated. Consistent with this, we observed that the seeds levitated close to the stable levitation position of a water droplet.

The polar auxin distribution in the root tips of seedlings in the 0 g* tube is altered, compared to samples in both the 1 *g* control outside the magnet, and the 1 g* control within the magnet. This shows that levitation has an effect on the distribution of auxin, in addition to the magnetic field effect discussed above. A similar alteration of the polar auxin distribution has also been found under simulated microgravity using the RPM [59]. This indicates that one effect of the microgravity environment is the accumulation of auxin in the root meristem. Our results show that this effect is independent of the movement of starch-rich statoliths. The increase in the amount of auxin could explain the increased proliferation rate observed under microgravity conditions.

The alteration of parameters related to cell growth and proliferation in these experiments follow the same trend that we observed in previous experiments carried out in real and simulated microgravity conditions (ISS and RPM, [17,22]). All data indicate that the root meristematic cells were capable of detecting the changes in the gravity vector by one or more mechanisms independent of the gravitropic response. The existence of mechanisms of perception of weightlessness by cells not containing apparent structures specialized in gravity sensing, has been proposed previously [60]. Our results indicate a connection between the response to the alteration of gravity, and auxin transport and metabolism; this connection was postulated elsewhere [61]. An example of these mechanisms would be gravity resistance or graviresistance, by means of which plant organs develop rigid structures with the purpose of preserving their proper location, resisting the gravitational force of attraction towards the centre of the Earth. In this mechanism, there is no need of cells specialized in gravity sensing, since this takes place by means of mechanoreceptors located in the plasma membrane (mechanosensitive ion channels) [62].

Samples grown in the 0 g* tube within the magnet showed an enhanced rate of cell proliferation accompanied by a reduction in ribosome biogenesis, compared to the controls in the 1 *g* and 1 g* tubes. This indicates that the strong magnetic field with a large vertical field gradient inside the 0 g* tube was capable of decoupling cell proliferation and ribosome biogenesis, and thus disrupting meristematic competence [17,22]. Similar results were observed in real microgravity during the “ROOT” experiment on board the ISS (“Cervantes” Spanish Soyuz Mission, October, 2003) and also in simulated microgravity conditions in a RPM [17]. Interestingly, we found a decrease in cyclin B1 gene expression associated with the increase in cell proliferation. This result is opposite to what is expected, since in ground conditions, cyclin B1 protein plays an essential role in the G2/M transition, and is thus used as a marker of cell proliferation [63]. Conversely, the accumulation of cyclin B1 observed in the mutant *tonsoku* was related to a prolongation of the G2 phase caused by activation of cell cycle-arresting proteins [64].

Our experiment suggests that meristematic cells exposed to simulated microgravity divide faster than in control conditions. Consequently, these cells are not able to reach their standard size and thus appear smaller and more numerous than in controls. These observations indicate that microgravity alters cell cycle regulation, leading to the shortening of the growth periods of interphase. Specifically, since the expression of the cyclin B1 gene takes place during the G2 phase and ribosome biogenesis peaks in this phase, the measurements of these two parameters are compatible with a shortening of G2. Previous experiments, using different ground-based simulation techniques [17,65], corroborate the interpretation of the uncoupling between cell growth and proliferation due to alterations in the regulation of the phases of the cell cycle.

The decrease in cell growth in root meristematic cells was assessed by observing the morphometric parameters of the nucleolus and the *in situ* concentrations of the nucleolar protein, nucleolin. The nucleoli of cells grown in the 0 g* tube exhibited the same general pattern of distribution of structural subcomponents corresponding to a normal meristematic cell, consistent with active ribosome production [30,31]. However, the smaller size of the nucleoli, the reduced granular component, and also the lower concentration of nucleolin, support the conclusion of a lower efficiency in the synthesis of ribosomes, as an effect of the simulated microgravity environment within the magnet, compared to the external control. We note, however, that the effect of magnetic levitation on this process is milder than in the case of real microgravity in space (ISS) or the simulated microgravity obtained in the RPM [17].

The effects observed in samples exposed to 2 g* were less pronounced than those observed in samples from the 0 g* tube. However, it is difficult in this case to discriminate which effects were due to gravity alteration and which effects were due to magnetic field ‘alone’ (i.e. 1 g* conditions, a magnetic field without the gradient needed to produce the diamagnetic force): on the one hand we observed similar but weaker alterations in samples exposed to magnetic field with no vertical field gradient (1 g*), but on the other hand, the data resembled those obtained from seedlings grown in a centrifuge rotating at 2 *g*[43]. Centrifugation produces some alterations in cell growth and proliferation which are opposite to the effects observed in real or simulated (RPM) microgravity [17]. In addition to this, a significant increase of the root meristem thickness was detected in samples from the 2 g* tube. The same effect has been observed under *2 g* centrifugation [43] (Additional file 1: Figure S1). This effect could be the consequence of modifications in the cell wall, which would become shorter, thicker and more rigid due to the accumulation of xyloglucans, to counteract the additional gravitational force [66].

## Conclusions

The residual effective gravitational forces acting on the plant when levitating are subtly different from those aboard the International Space Station, for example. In these experiments, the cellular components with a magnetic mass susceptibility close to that of water were levitated by the magnetic field. These materials include the bulk of the solid cellular material, the intracellular water and water-soluble cellular components, but, importantly for these experiments, do not include the starch-rich statoliths within the cells. The whole plant was levitated by the magnetic field in these experiments, reducing the gravitational stresses on the plant, but the field we used did not disrupt statolith sedimentation, and therefore the gravitropism mechanism is preserved unaltered. The changes we see in cell growth and proliferation in the root meristem of levitated seedlings are comparable with changes observed in systems lacking known mechanisms for detection of gravitropic signals (e.g. cell cultures), which, interestingly, show similar alterations in cell growth and proliferation. The alterations observed are similar to those found in real microgravity aboard the ISS, or mechanically simulated microgravity (RPM). The special environment of levitation has allowed us to observe that alterations in these processes caused by gravity changes are independent of statolith movement, while still related to auxin distribution. This suggests a redundancy in the mechanisms of gravitropic response and gravity resistance in plants. We also found that the strong magnetic field without a vertical field gradient influences auxin polar transport in the root tips of the seedlings, but that a greater influence is observed on seedlings grown under levitation, i.e. in a magnetic field with a large vertical field gradient.

## Authors’ contributions

AIM, closely supported by RH, carried out the experiments in the magnetic field, performed sample processing, molecular genetic studies and data analyses, and drafted the manuscript. OJL, CED designed the experimental apparatus for the magnet system and carried out the experiments in the magnet. OJL constructed the apparatus. RJAH developed the experimental apparatus with OJL, performed the calculations of the magnetic field and effective gravity, operated and advised on the use and effects of the magnet system, and edited the manuscript. PA, MRD, LE, and mainly FJM, participated in the study design and coordination and helped to draft and edit the manuscript. FJM conceived the study. RJAH and LE were responsible for establishing the magnetic levitation laboratory in Nottingham. All authors read and approved the final manuscript.

## Supplementary Material

Additional file 1: Figure S1 Macroscopic morphometric parameters of the seedlings. The four panels, corresponding to samples from the 1 *g* control, 0 g*, 1 g* and 2 g* tubes within the magnet, show the root width in the central, semithin, sections of the root tip, after 2 days’ growth. The measurements were obtained from microscope images. Click here for file
