# Supplementary figures and images for: Meristematic cell proliferation and ribosome biogenesis are decoupled in diamagnetically levitated Arabidopsis seedlings
Source: BMC Plant Biol. 2013 Sep 5;13:124. doi: 10.1186/1471-2229-13-124 (PMC3847623; doi:10.1186/1471-2229-13-124)

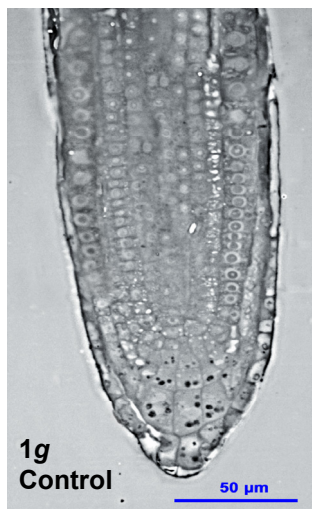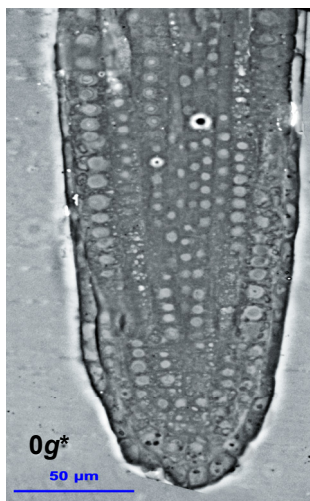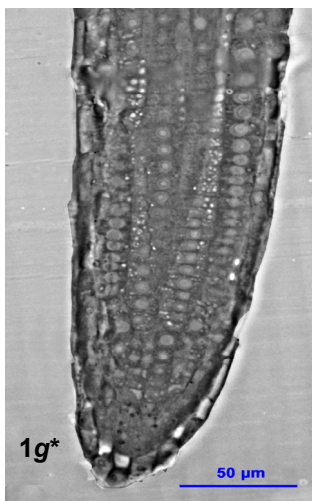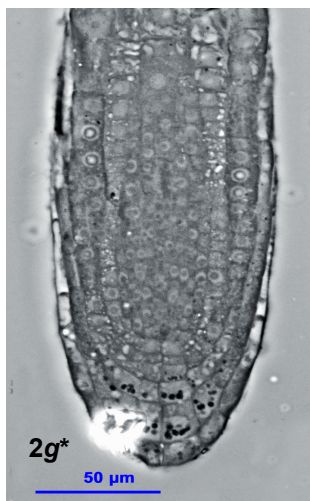

Supplement: Additional file 1: Figure S1 — Macroscopic morphometric parameters of the seedlings. The four panels, corresponding to samples from the 1 g control, 0 g*, 1 g* and 2 g* tubes within the magnet, show the root width in the central, semithin, sections of the root tip, after 2 days’ growth. The measurements were obtained from microscope images. [file 1471-2229-13-124-S1.pdf]
